# Thickness Characterization Toolbox for Transparent Protective Coatings on Polymer Substrates

**DOI:** 10.3390/ma11071101

**Published:** 2018-06-28

**Authors:** Matthias Van Zele, Jonathan Watté, Jan Hasselmeyer, Hannes Rijckaert, Yannick Vercammen, Steven Verstuyft, Davy Deduytsche, Damien P. Debecker, Claude Poleunis, Isabel Van Driessche, Klaartje De Buysser

**Affiliations:** 1 Sol-Gel Centre for Research on Inorganic Powders and Thin Film Synthesis (SCRiPTS), Department of Chemistry, Ghent University, Krijgslaan 281-S3, 9000 Ghent, Belgium; Matthias.VanZele@ugent.be (M.V.Z.); Jonathan.Watté@ugent.be (J.W.); Jan.Hasselmeyer@ugent.be (J.H.); Hannes.Rijckaert@ugent.be (H.R.); Isabel.VanDriessche@ugent.be (I.V.D.); 2 Department of Chemistry, Universiteit Antwerpen, Campus Drie Eiken, Universiteitsplein 1, 2610 Wilrijk, Belgium; mail@yannickv.be; 3 Photonics Research Group, Department of Information Technology, Ghent University-IMEC, 9000 Ghent, Belgium; Steven.Verstuyft@ugent.be; 4 Conformal Coating of Nanomaterials (CoCooN), Department of Solid State Sciences, Ghent University, Krijgslaan 281-S1, 9000 Ghent, Belgium; davy.deduytsche@ugent.be; 5 Institute of Condensed Matter and Nanosciences, Université catholique de Louvain, 1348 Louvain-la-Neuve, Belgium; damien.debecker@uclouvain.be (D.P.D.); claude.poleunis@uclouvain.be (C.P.)

**Keywords:** coating materials, inorganic materials, surfaces and interfaces, sol-gel processes, low-emissivity, SIMS

## Abstract

The thickness characterization of transparent protective coatings on functional, transparent materials is often problematic. In this paper, a toolbox to determine the thicknesses of a transparent coating on functional window films is presented. The toolbox consists of a combination of secondary ion mass spectrometry and profilometry and can be transferred to other transparent polymeric materials. A coating was deposited on designed model samples, which were characterized with cross-sectional views in transmission and in scanning/transmission electron microscopy and ellipsometry. The toolbox was then used to assess the thicknesses of the protective coatings on the pilot-scale window films. This coating was synthesized using straightforward sol-gel alkoxide chemistry. The kinetics of the condensation are studied in order to obtain a precursor that allows fast drying and complete condensation after simple heat treatment. The shelf life of this precursor solution was investigated in order to verify its accordance to industrial requirements. Deposition was performed successfully at low temperatures below 100 °C, which makes deposition on polymeric foils possible. By using roll-to-roll coating, the findings of this paper are easily transferrable to industrial scale. The coating was tested for scratch resistance and adhesion. Values for the emissivity (ε) of the films were recorded to justify the use of the films obtained as infrared reflective window films. In this work, it is shown that the toolbox measures similar thicknesses to those measured by electron microscopy and can be used to set a required thickness for protective coatings.

## 1. Introduction

Thickness effects of transparent thin coatings often seems to be a critical factor in the design of functional devices. These coatings should be thick enough to protect the underlying materials. On the other hand, losses in transparency can be noticed with an increased thickness, which is often detrimental for their application.

This paper looks into the field of insulating window films. These window films are designed in such a way that heat is kept outside of buildings during summer times and vice versa. This functionality is achieved by the deposition of a far infrared reflective Fabry-Pérot stack with low emissivity (ε) on polymer films. The Fabry-Pérot stack consists of transparent layers, deposited typically by subsequent sputtering. An example is a TiO_2_/Ag/TiO_2_ multi-layer stack. This stack shows excellent low-ε properties [1].

The aforementioned stack is often used outside the field of window films and placed in between two glazing panels, where no moisture can reach the stack. This is crucial, as it is well known that the inner metallic silver layers are not stable and that they are especially sensitive to moisture. Several reports have been published on the moisture-induced degradation of sputtered low-ε coatings containing silver, concluding that high concentrations of chlorides could lead to the corrosion of the stack [2,3,4,5,6,7]. Therefore, contact with moisture and chlorides is detrimental for the low-ε properties of these stacks and should be protected with an additional coating.

When looking outside the scope of flexible polymeric window films, many coatings have been reported to effectively protect this kind of stacks on solid substrates. One way of protecting stacks is to apply ceramic materials, such as ZnO, Al-doped ZnO [8], or Al_2_O_3_ material, as the final hard coat [6]. These ceramic coatings are often deposited by sputtering [9], which is not very cost-effective. Another approach is to use organic coatings to protect underlying layers and materials which are prone to mechanical damage [10,11]. These organic coatings, however, absorb far infrared radiation, which renders the reflecting stacks on window films useless, as the films will heat up and loss of heat reflection will occur. For the same reason, no hybrid materials containing scratch-resistant metal oxide particles, such as silica nanoparticles, embedded in an organic matrix [12,13,14] could be used for this application.

To overcome the drawbacks of all known protective coatings and thus preserve the low-ε characteristics of mentioned window films, a silica thin layer was deposited through a wet chemical deposition method. This protective coating should not only meet the requirements for industrial applications, but also should be cost-effective. Chemical solution deposition (CSD) is a cost-effective deposition method for thin films. CSD starts from molecules in solution or suspension, which are deposited on a surface, which results in the growth of a new phase. The method has proven to be effective to deposit a wide range of materials [8,15,16,17,18,19].

Industrial requirements for the protective coating include a class 0 adhesion and 3H pencil hardness, according to ASTM D3359 [20] and ASTM D3363 [21], respectively. Because of these goals, the possibility to deposit this coating by using roll-to-roll deposition processes was further explored.

The aim of this work is to develop a thickness determination toolbox for transparent protective coatings on transparent substrates and to assess the minimal thickness to pass the predefined application tests of a protective silica coating for low-ε window films. Ellipsometry, scanning, and transmission electron microscopy (SEM and TEM) are often the characterization techniques of choice. However, ellipsometry is not suitable for fully transparent devices containing a relatively thick, transparent substrate. This is because ellipsometry is not able to distinguish thin layers from the underlying substrate in transparent devices, as the difference in refractive indices between the layers is too low. Ellipsometry fails because the signal coming from the thin layers is shadowed by the signal of the substrate. Cross-sectional view SEM and TEM measurements, on the other hand, are very good techniques to determine the thickness of top coatings, but are not suitable if polymer substrates are present, as the electron beam burns through the organic substrates [22]. To circumvent these drawbacks, we propose to combine secondary ion mass spectrometry (SIMS) and profilometry as an effective thickness determination toolbox.

The idea behind this proposed toolbox is to use the SIMS apparatus to dig a crater in the film while checking the chemical composition of the surface (by analyzing the ions that are emitted). In this way, the sudden appearance of the ions attributed to the support is a direct indication that the upmost film has been eroded. The sputtering is then interrupted and the crater is analyzed by profilometry to determine its depth, which strictly corresponds to the thickness of the film.

**Figure 1:**
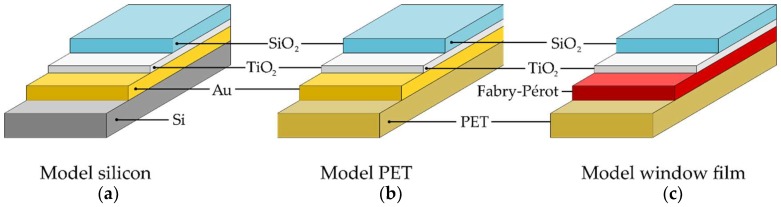
Composition of (**a**) the TiO_2_/Au/silicon model sample; (**b**) the TiO_2_/Au/polyethylene terephtalate (PET) model sample; (**c**) the window film. The model sample was built up on silicon or plain PET, on which a gold layer was deposited. The window film functionality comes forth from the functional metal/metal oxide layer. All samples were provided with a titania buffer layer, on which the protective silica coating was deposited.

To develop this toolbox, model samples were designed (Figure 1a,b), comparable to window films (Figure 1c). The model samples were characterized with ellipsometry, SEM/TEM, and the proposed SIMS-profilometry toolbox. An additional gold layer was sputtered to make ellipsometry and SEM/TEM measurements possible. On the one hand, the gold layer is needed to make a distinction between the individual layers with ellipsometry. On the other hand, this conducting gold layer is necessary to make Focused ion beam (FIB)-SEM measurements possible. Otherwise, too much drifting of the sample would occur as the samples become charged during measurement. In order to make TEM measurements possible, the polymer substrate was changed to silicon.

In this work, it is shown that the proposed toolbox delivers similar thicknesses to those observed in cross-sectional view SEM/TEM images, while ellipsometry shows different values of thickness. Electronic microscopy (EM) methods are considered to be accurate in the determination of film thickness. In order to check this hypothesis, both results obtained by the proposed toolbox and by ellipsometry are compared to EM. The conclusions drawn from model sample characterizations are transferred to comparable functional window films, which cannot be characterized by EM. It is shown that the proposed toolbox can be used to determine the thicknesses of thin coatings on transparent polymeric substrates.

## 2. Materials and Methods

The chemicals were used as received. Absolute ethanol was obtained from Applichem Panreac (Darmstadt, Germany). Hydrochloric acid (36 %) and glacial acetic acid (100 m%) were obtained from Roth (Karlsruhe, Germany). The silane precursor tetraethyl orthosilicate (TEOS, 99% pure) was supplied by ABCR (Karlsruhe, Germany). Deuterated water was obtained from Sigma Aldrich (Overijse, Belgium).

Sample fabrication: A graphical representation of sample compositions can be seen in Figure 1. The substrate used as model sample was either silicon (1 × 1 cm^2^) (Figure 1a) or plain PET (1 × 1 cm^2^) (Figure 1b), provided with a gold layer and a titania buffer layer, both deposited by physical vapor deposition. The 100-nm thick gold layer was deposited using electron beam operation with a base pressure of 1.0 × 10^−6^ mbar. Also, the 5-nm thick TiO_x_ layer was deposited using the electron beam operation of Ti, under an oxygen partial pressure of 4.0 × 10^−5^ mbar.

Window films (Figure 1c) were provided by Group Michiels Advanced Materials (M.A.M.) and consisted of a 20-cm wide and 50-µm thick PET film. This PET film was functionalized with a single sputtered Fabry-Pérot stack (Zele, Belgium). On top of this stack, a titania buffer layer was sputtered.

Coating precursor synthesis: A sol-gel synthesis was adapted from previous work [15,23,24,25]. In a typical synthesis procedure, tetraethyl orthosilicate was added to ethanol. In a separate container, a solution of glacial acetic acid in distilled water was prepared. Acetic acid is needed in order to catalyze the condensation reaction. All reagents were mixed while stirring. An additional pre-condensation step was conducted by heating the total mixture for 3 h under refluxing conditions at 60 °C. As a final step, the reaction was quenched with ethanol. Four hundred milliliters of a transparent sol was obtained in accordance with patent EP15199592.5 [26].

Coating deposition: TiO_2_/Au/silicon and TiO_2_/Au/PET model substrates were coated using spin-coating by dropping 90 µL of the transparent sol on the substrate. The sample was then spun for 60 s at 2000 rpm with a spin acceleration of 40 rpm/s^2^. After spin-coating, it was annealed at 80 °C for 60 s on a hot plate in an ambient atmosphere.

**Figure 2:**
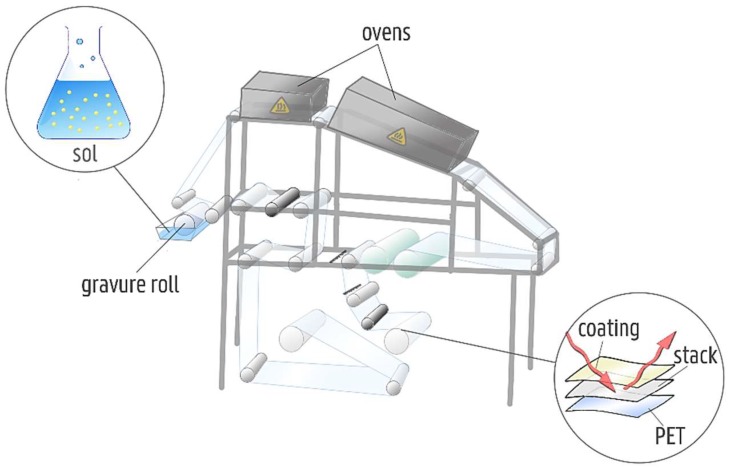
Schematic overview of a roll-to-roll coater. The foil is transported by multiple rolls, while deposition of the sol is achieved by a gravure roll. Before rewinding, a heat treatment takes place in the top ovens.

Window film substrates were coated via roll-to-roll coating (Figure 2), using the coating conditions summarized in Table 1. Characterized window films were coated once and coated twice using these coating settings.

Raman characterization: The kinetics of the pre-condensation step were monitored with a RamanRxn system from Kaiser optical systems (Ann Arbor, MI, USA), model Rxn1-532. The measurements were performed in situ by placing a laser probe inside the reaction mixture. The mixture was put in a three-neck flask, equipped with a reflux cooler and a dropping funnel to add reagents, together with the laser probe. The flask, the dropping funnel, and the reflux cooler were covered in aluminum foil to prevent the detection of radiation coming from the outside of the mixture. The wavelength of the laser was 532 nm with a maximum power output of 450 mW. A resampling interval of 1 cm^−1^ was used while recording 13 accumulations with an exposure time of 10 s.

Nuclear Magnetic Resonance (NMR): To evaluate the degree of condensation and the correlated shelf life of the sols, ^29^Si-NMR measurements were performed using a Bruker (Billerica, MA, USA) Avance III spectrometer with a 1 h frequency of 500 MHz, equipped with a 5-mm BBI Z probe. Samples were prepared by adding 100 µL of D_2_O to 900 µL of the sol before transferring to an NMR test tube.

Spectroscopic characterization: Optical transmission and reflection spectra were recorded with an Agilent Cary 5000 UV-Vis-NIR spectrophotometer (Santa Clara, CA, USA). The spectra were recorded from 300 to 2000 nm.

Application Tests: Crosshatch tests were performed according to ASTM D3359 standard [16]. A cutter size of 1 mm was used with six cutting edges. The adhesion test tape has an adhesive strength of 9.5 N per 25-mm width. Both were supplied by Paint Test Equipment (Congleton, UK). The sample was prepared by a first cut in the coating with the cutter. A second cut, perpendicular to the first cut was made. The adhesive tape was placed over the damaged area and, finally, the tape was removed from the surface. Using a magnifying glass, the coating was checked for damage.

The hardness of the coating was evaluated with an Elcometer (Utrecht, The Netherlands) 501 Pencil Hardness Tester. The method used was the Wolff-Wilborn method. The equipment complies with the ASTM D3363 standard [17].

The emissivity ε was measured with a TIR 100-2, supplied by Inglas (Friedrichshafen, Germany). The samples were cut to a size of 10 by 10 cm.

Layer thickness: The proposed toolbox should be compared to other layer thickness characterization techniques to verify its effectiveness. The cross-sectional view images and lamella were prepared by a FEI Nova (Hillsboro, OR, USA) 600 Nanolab Dual-Beam Focused Ion Beam system and associated SEM.

For TEM analysis, a cross-sectional lamella was obtained using ion milling techniques via the FIB in situ lift-out procedure with an Omniprobe (FEI, Hillsboro, OR, USA) extraction needle and top cleaning. High-angle annular dark-field (HAADF) and bright-field (BF) scanning TEM images were taken on a JEOL JEM-2200FS TEM (Tokyo, Japan) with a Cs corrector, operated at 200 kV. Chemical information was obtained via the phase analysis via energy dispersive X-ray spectroscopy.

Spectroscopic ellipsometry measurements were performed using a J.A. Woollam M-2000 ellipsometer (J.A. Woollam Co., Lincoln, NE, USA). The spectral range of the ellipsometer ranged from 250 to 1680 nm and the COMPLETEEASE software (version 6.34) was used for fitting and data analysis.

Ellipsometry mapping measurements were performed on a homemade mapping stage. The nominal angle of incidence for all measurements was fixed at 70°. The acquisition time for one spectrum was set at 1.5 s.

The measured data was modeled using a B-spline layer for the Au film, a Cauchy layer for the TiO_2_ substrate, and another Cauchy layer for the silica film. The thicknesses of the various layers were determined using fixed parameters for the Cauchy parameters.

Depth profiles SIMS were made using a TOF.SIMS5 (IONTOF GmbH, Münster, Germany) time-of-flight secondary ion mass spectrometer [27]. This instrument is equipped with a Cs+ ion beam as a sputtering source and a liquid metal ion gun (Bi) as an analytical source, both mounted at 45° with respect to the sample surface. The time-of-flight mass analyzer is perpendicular to the sample surface. Depth profiles were carried out in the ‘interlaced’ mode in a cycle time of 100 µs. During this cycle time, the analytical beam (pulsed Bi_5_^+^ for SIMS analysis) was followed by periods of continuous Cs^+^ ion sputtering. Low energy electrons were also sent to the sample during this cycle in order to recover the initial surface potential. The Cs^+^ ion source was operated at 500 eV with a direct current of 40 nA. For depth profiling, the focused Cs^+^ beam of primary ions was rastered over an area that typically measured 450 × 450 μm^2^. A pulsed beam of 30 keV Bi_5_^+^ (alternating current of 0.11 pA) ions was employed to provide mass spectra from a 150 × 150 μm^2^ area in the center of the sputter crater. Charge compensation was conducted using an electron flood gun (*E*_k_ = 20 eV). All data analyses were carried out using the software supplied by the instrument manufacturer, SurfaceLab (version 6.5). Depth profiling was stopped when the Ti^+^ signal reached 50% of its maximum intensity.

Stylus profilometer was used to measure the craters obtained by SIMS measurements (DektakXT, Bruker Nano Surfaces Division, Tucson, AZ, USA). The stylus had a radius of 0.7 µm and the applied force was 0.1 mg. Four line scans were conducted over the measured crater in order to obtain average and standard deviation measurements.

## 3. Results and Discussion

### 3.1. Precursor Synthesis and Deposition

Keeping industrial upscaling in mind, one must be able to produce a coating precursor in a relatively short time-span, which can be conserved for longer times. Raman spectroscopy has proven to be a valuable technique to monitor the condensation species of silicon-based sol-gel reactions [28,29,30]. This technique was used to follow the condensation reaction over time.

**Figure 3:**
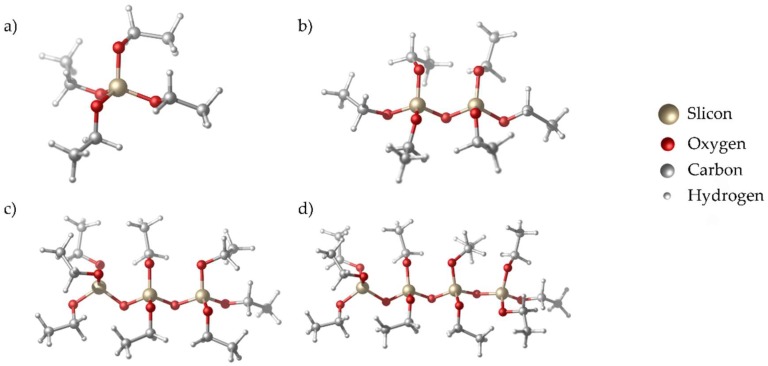
Graphical representation of the species monitored with Raman spectroscopy. The monomer species (**a**) reacts subsequently to dimer (**b**), trimer (**c**), and tetramer (**d**) species.

**Figure 4:**
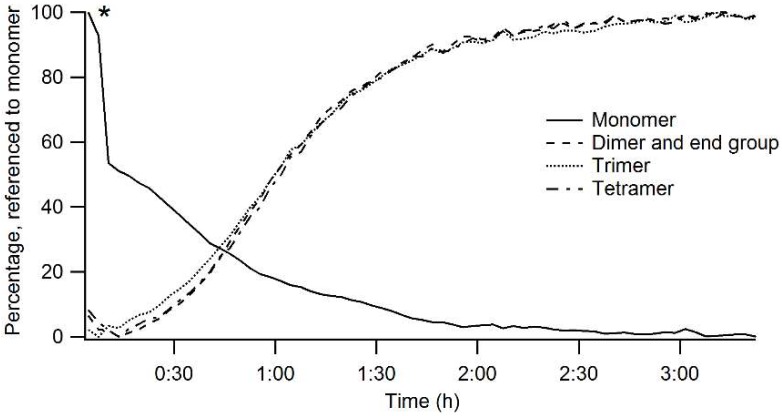
Raman intensities monitored during the pre-condensation step. All signals are referenced to the monomer. The marked (∗) drop in intensity shows the addition of the catalyst, diluted in water.

Figure 3 shows a graphical representation of the monitored species. Table 2 describes the bands that are monitored. As can be seen, chain length is the main characteristic that is monitored with Raman spectroscopy. Figure 4 shows the Raman intensities of monomer, dimer (including end groups), trimer, and tetramer species present in the reaction mixture when using acetic acid as a catalyst. The marked drop in intensity of the monomer signal indicates the addition of catalyst and water, which are necessary for the condensation reaction. After the addition, a rise in dimer, trimer, and tetramer was recorder. This means that the sol starts condensing immediately after the addition of the diluted catalyst. What is more important is the time the reaction mixture takes to reach an equilibrium state. As can be seen in Figure 4, after 3 h almost all monomer species were depleted and converted into more condensed species.

**Figure 5:**
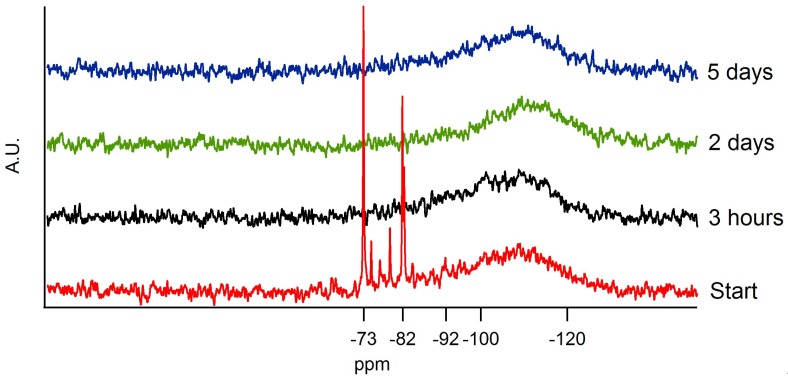
NMR spectrogram of the sol at different time intervals.

Once the reaction mixture reaches this equilibrium state, it is important to inhibit further condensation. In order to obtain this inhibition, a quenching step is induced by the addition of ethanol. To map the stability of the sol at longer times after this quenching step, ^29^Si-NMR was used. ^29^Si-NMR analyses show how many times an ethoxy group is substituted by another siloxane group and thus the condensation degree. The species monitored are shown in Table 3. The spectra obtained are shown in Figure 5. Immediately after the addition of the diluted catalyst, the spectrogram labeled ‘Start’ was recorded. Some resonances are of particular interest. The resonance at −73 ppm corresponds to the four-times hydrolyzed species Q^0^_hydrolized_, with formula Si(OH)_4_. Furthermore, −82 ppm corresponds to non-hydrolyzed TEOS monomer. The resonances between −73 and −82 ppm are due to three-, two-, and one-time hydrolyzed monomer units. The resonance of the one-time condensed species Q^1^ could not be distinguished from the monomer at −82 ppm. The resonance at −92 ppm stands for the two-times condensed TEOS, labeled as Q^2^. The region between −100 and −120 ppm reflects the three-times and four-times condensed species, Q^3^ and Q^4^, respectively [30,31,32]. Here, no clear distinction between three-times and four-times condensed species is possible.

Figure 5 shows the ^29^Si-NMR spectrum at the start of the condensation step. Q^0^_hydrolized_ species are distinguishably present at the start and are converted into other, more condensed species over time. The region between −73 and −82 ppm shows four resonances, corresponding to one-, two-, three-, and four-times hydrolyzed silicon atoms. It can be seen that already a fraction of Q^3^ and Q^4^ is detected.

After a condensation of 3 h and subsequent quenching, all hydrolyzed species are converted and a shift towards Q^3^ and Q^4^ can be noted.

^29^Si-NMR measurements at longer gelation times were performed to monitor the shelf-life of the sol. Three possible hypotheses were set up before the experiment. The first one indicated that the sol would be stable in time. No significant change should be noticeable in the ^29^Si-NMR spectrograms. The second hypothesis depicted the condensed material being broken up by the excess amount of ethanol present in the reaction mixture. The intensities in the region between −100 and −120 ppm should lower and shift to higher ppm values. A last hypothesis depicted the total condensation of all the species. For this hypothesis to be true, the intensities between −100 and −120 ppm should shift to lower ppm values. As can be seen in Figure 5, no significant change in the region of interest was observed. A very small shift towards −120 ppm was noticed after which an equilibrium was found. This equilibrium implies that the sol is still usable for coating processes after longer times.

The aforementioned silica precursor was deposited both on the model samples and window films.

### 3.2. Layer Thickness Determination

The characterization of the coating thickness was carried out by three independent techniques, namely EM, ellipsometry, and the proposed characterization toolbox, defined as a combination of SIMS with profilometry. The results are listed in Table 4. EM is reported to be the technique of choice for thickness characterization, while ellipsometry is often used when EM is not possible. When looking at the results in Table 4, one can conclude that the values for EM and ellipsometry do not correspond well to each other. This is because of the high complexity of the model sample. Ellipsometry is not able to distinguish the top thin layer from the underlying interlayers and the substrate, as it is a technique that needs a significant difference in refractive index between two layers to make a distinction. As this difference is not present prominently in window films between the top coating and the substrate, ellipsometry measurements are shadowed by the relatively thick substrate. Even the sputtering of an additional gold layer as an interface does not lead to conclusive ellipsometry measurements.

**Figure 6:**
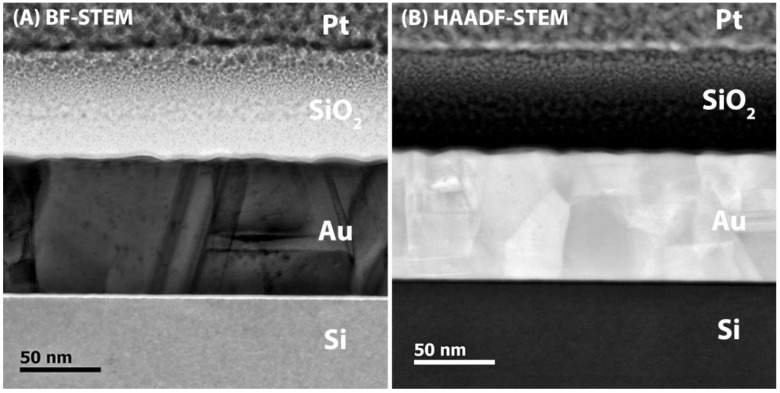
Cross-sectional (**A**) BF- and (**B**) HAADF-STEM image of TiO_2_/Au/silicon model sample a. Individual layers can be distinguished by using Z-contrast.

**Figure 7:**
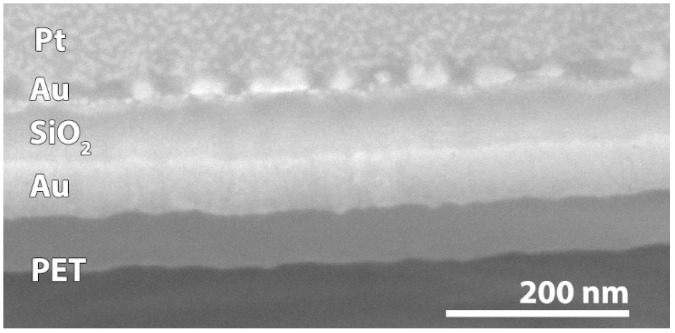
Cross-sectional SEM image of TiO_2_/Au/PET model sample b.

When comparing the proposed toolbox to EM measurements on the model substrates (Figure 6 and Figure 7), the results match closely. Thus, the combination of SIMS and profilometry can offer a possibility to determine the thickness of the top coating, as long as the interface between the top coating and substrate is detectable. As SIMS envelops mass spectrometry, this technique can detect individual ions. Therefore, SIMS shows the possibility to distinguish different layers as soon as the atomic composition is different, meaning that other ions are present in each layer or that the relative concentration differs between layers.

**Figure 8:**
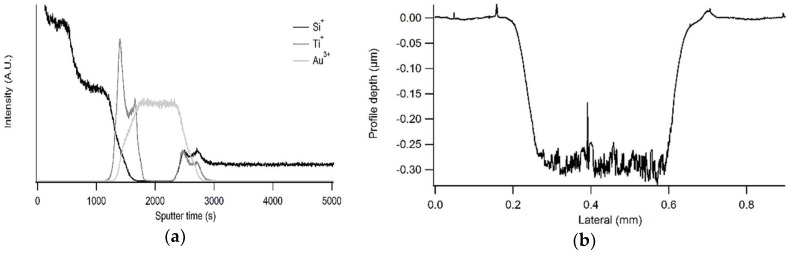
Complete profile of Si model sample b obtained with SIMS (**a**). Profilometry analysis (**b**) was conducted on a fresh zone and sputtering was stopped when the Ti signal reached half of the maximal intensity.

Figure 8a shows a representative SIMS depth profile obtained on silicon model substrate b. To measure the crater depth, a fresh zone was selected and sputtering was stopped at the interface between the protective coating and the TiO_2_ buffer layer. This was detected by a steep increase of Ti^+^ ions. The depth of the sputtered crater was then determined using profilometry (Figure 8b). As can be seen in Table 4, coating thicknesses were determined to be 126 ± 8 nm for single coated window films and 274 ± 12 nm for double coated window films by the proposed toolbox.

### 3.3. Window Film Characteristics

#### 3.3.1. Application Tests

**Figure 9:**
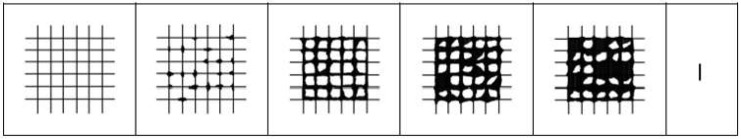
Crosshatch classification. On the left-hand side class 0 is depicted. On the right-hand side class 4 is depicted. Class 5 corresponds to a complete removal of the coating.

The coating deposited on window films was tested for adhesion, ASTM D3359 [20], and scratch resistance, ASTM D3363 [21]. To evaluate the adhesion of the layer, the standard provides a classification to compare the sample. Class 0 relates to very good adhesion, while class 5 relates to very poor adhesion. The classification is depicted in Figure 9. The scratch resistance standard used is a straightforward technique where pencils with varying hardness are used to scratch the sample.

The double coated sample was able to withstand scratch attempts with a 3H pencil and showed a class 0 adhesion, using a simple crosshatch test. The single coated film, however, also showed class 0 adhesion but was not able to pass the pencil hardness test using a 3H pencil. One can say that a coating thickness of 274 ± 12 nm is needed to obtain scratch-resistant coatings with good adhesion. The minimal required thickness could be lower, but this falls out of scope of this work.

#### 3.3.2. Visual Transmission and Infrared Reflection Properties

To be able to use a window film onto which a heat-reflecting layer is applied, good visual transparency and optimal infrared reflectivity should be achieved. UV-VIS-IR measurements were recorded in transmission and reflection modes.

**Figure 10:**
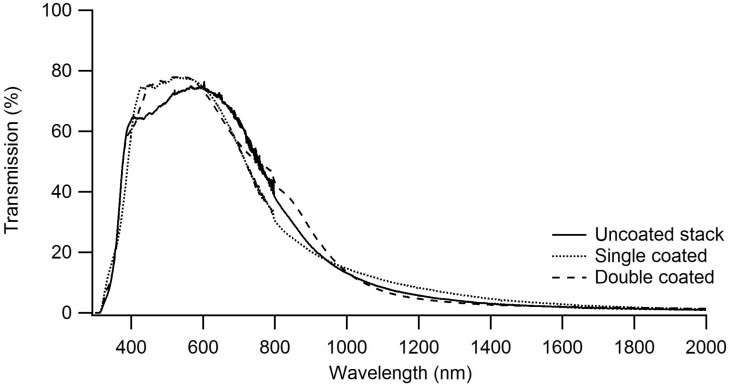
Transmission spectra of the uncoated stack and samples of single coated window film and double coated window film. The double coated window film is more reflective in the region between 400 nm and 600 nm than the single coated window film.

**Figure 11:**
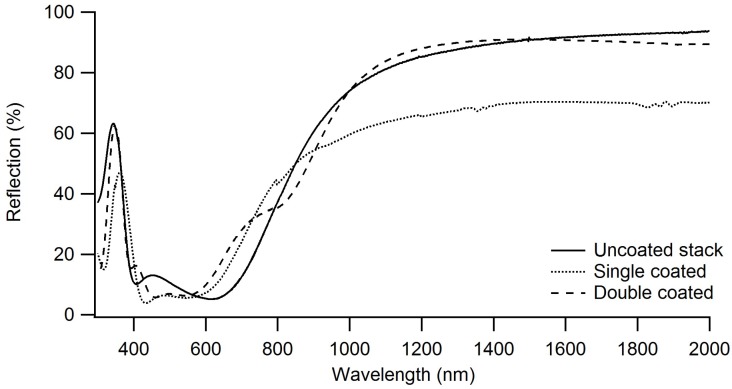
Reflection spectra of the uncoated stack, single coated window film, and double coated window film. The double coated is more reflective than single coated film at higher wavelengths.

As shown in Figure 10, there is a difference in visual transparency between the uncoated stack and coated samples in the region between 400 and 600 nm. Visual transparency increases when applying the silica coating, while there is almost no difference between the two coated samples. This is not the case for the reflection spectra, as shown in Figure 11; almost no difference is seen between the uncoated stack and double coated film, while a big difference is noted between the uncoated stack and single coated film on the one hand and double coated film on the other hand between 800 and 2000 nm. This drop is not present for the double coated window film.

It is thus shown that the silica layer interferes with the Fabry-Pérot stack in such a way that higher visual transparency is obtained, while maintaining the heat reflecting properties. The fact that this changes with coating thicknesses proves that this is due to the combination of refractive indices of the silica coating and the Fabry-Pérot stack.

These findings were confirmed by the emissivity values, which ranged from 0.022 for the uncoated stack to 0.031 for the double coated film. The last value is well below the threshold of 0.1 for heat-reflecting window films.

## 4. Conclusions

The main conclusion of this work is that the proposed thickness characterization toolbox for transparent protective coatings on transparent devices is proven to be a valuable method. The results obtained by this combination of SIMS and ellipsometry are in agreement with those achieved using electron microscopy for the designed model samples. The toolbox was able to determine the thicknesses of thin layers deposited on functional window films, where other techniques failed.

This research also showed synthesis and deposition methods for the large-scale application of scratch-resistant coatings on polymeric, heat-reflecting window films. Both Raman and ^29^Si-NMR spectroscopy were used to assess precursor synthesis and the following shelf-life. The results showed that an optimal coating precursor was obtained after 3 h of condensation and that a shelf-life up to five days was guaranteed. After simple and straightforward coating deposition with roll-to-roll coating, application tests were conducted. It was shown that a coating thickness of 274 ± 12 nm leads to class 0 adhesion, 3H pencil hardness resistance, optimal visual transparency, and an even improved infrared reflectivity, compared to uncoated samples. It is possible that a lower coating thickness would be sufficient, keeping in mind the lower threshold of 126 ± 8 nm, but this was not studied further. By avoiding use of chloride containing reagents, further scaling up towards industrial settings is possible. It is proven that this chloride-free synthesis has no negative effects on the drying and curing of the coating, as the coating passed all application tests. A low temperature heat-treatment program was shown to be sufficient to obtain these scratch-resistant coatings. A maximum temperature of 80 °C was needed to fully dry and cure the coating. It is thus possible to transfer these results to other temperature-sensitive substrates.

In short, this research demonstrated an effective thickness characterization toolbox. Additionally, during this research an easily up-scalable coating was developed. This coating is intended to protect scratch-sensitive substrates, which cannot be coated with organic or hybrid state-of-the-art materials, due to their optical properties.

## 5. Patents

The protective coating precursor composition was patented under number EP15199592.5 [26].

## Figures and Tables

**Table 1:** Coating settings for roll-to-roll coating.

Parameter	Setting
Unwinding speed	2.4 m/min
Application speed	1 m/min
Coating method	Reverse gravure
Temperature first oven	60 °C
Temperature second oven	80 °C

**Table 2:** Description of the bands monitored by Raman spectroscopy [30].

Band Profile	Description
Monomer	Height to zero, peak from 665 to 645nm
Dimer and end group	Height to zero, peak from 605 to 585 nm, referenced to monomer
Trimer	Height to zero, peak from 580 to 560 nm, referenced to monomer
Tetramer	Height to zero, peak from 560 to 540 nm, referenced to monomer

**Table 3:** ^29^Si chemical shifts and corresponding silicate structures [30].

Label	δ ppm	Silicate Structure
Q^0^_hydrolized_	−73	Si*(OH)_4_
Q^0^	−82	Si*(OR)_4_
Q^2^	−92	Si*(OR)_3_(−O−Si≡)
Q^3^ and Q^4^	−100 to −120	Si*(OR)_2_(−O−Si≡)_2_ Si*(OR)(−O−Si≡)_3_

R corresponds to C_2_H_5_. Each peak corresponds to the marked silicon atom (*)

**Table 4:** Results overview of secondary ion mass spectrometry (SIMS), electronic microscopy (EM), and ellipsometry measurements. For each model type, two individual samples (a and b) were synthesized and characterized. A graphical representation is shown in Figure 1.

Model Substrate	SIMS (nm)	EM (nm)	Ellipsometry (nm)
TiO_2_/Au/silicon (a)	–	63.9	88.2
TiO_2_/Au/silicon (b)	63.6 ± 5.7	61.8	92.9
TiO_2_/Au/PET (a)	61.0 ± 6.3	57.5	95.2
TiO_2_/Au/PET (b)	84.7 ± 8.2	82.8	77.8
Window film (a)	126 ± 8	X	X
Window film (b)	274 ± 12	X	X

All values are noted in nm. X denotes that the measurement was not conclusive.
